# Evolution of the TGF-β Signaling Pathway and Its Potential Role in the Ctenophore, *Mnemiopsis leidyi*

**DOI:** 10.1371/journal.pone.0024152

**Published:** 2011-09-08

**Authors:** Kevin Pang, Joseph F. Ryan, Andreas D. Baxevanis, Mark Q. Martindale

**Affiliations:** 1 Kewalo Marine Laboratory, Pacific Biosciences Research Center, University of Hawaii at Manoa, Honolulu, Hawaii, United States of America; 2 Genome Technology Branch, National Human Genome Research Institute, National Institutes of Health, Bethesda, Maryland, United States of America; University of Otago, New Zealand

## Abstract

The TGF-β signaling pathway is a metazoan-specific intercellular signaling pathway known to be important in many developmental and cellular processes in a wide variety of animals. We investigated the complexity and possible functions of this pathway in a member of one of the earliest branching metazoan phyla, the ctenophore *Mnemiopsis leidyi*. A search of the recently sequenced *Mnemiopsis* genome revealed an inventory of genes encoding ligands and the rest of the components of the TGF-β superfamily signaling pathway. The *Mnemiopsis* genome contains nine TGF-β ligands, two TGF-β-like family members, two BMP-like family members, and five gene products that were unable to be classified with certainty. We also identified four TGF-β receptors: three Type I and a single Type II receptor. There are five genes encoding Smad proteins (Smad2, Smad4, Smad6, and two Smad1s). While we have identified many of the other components of this pathway, including Tolloid, SMURF, and Nomo, notably absent are SARA and all of the known antagonists belonging to the Chordin, Follistatin, Noggin, and CAN families. This pathway likely evolved early in metazoan evolution as nearly all components of this pathway have yet to be identified in any non-metazoan. The complement of TGF-β signaling pathway components of ctenophores is more similar to that of the sponge, *Amphimedon*, than to cnidarians, *Trichoplax*, or bilaterians. The mRNA expression patterns of key genes revealed by *in situ* hybridization suggests that TGF-β signaling is not involved in ctenophore early axis specification. Four ligands are expressed during gastrulation in ectodermal micromeres along all three body axes, suggesting a role in transducing earlier maternal signals. Later expression patterns and experiments with the TGF-β inhibitor SB432542 suggest roles in pharyngeal morphogenesis and comb row organization.

## Introduction

The transforming growth factor-β (TGF-β) signaling pathway was first discovered about 30 years ago, a pathway in which certain secreted proteins had the capability of transforming cells and tissues. The first TGF-β gene was cloned in 1985 [1]. Since then, similar proteins were discovered in animals as diverse as flies, nematodes, and vertebrates, all of which had similar functions in tissue morphogenesis (reviewed in [2]–[5]). Through the use of cloning and sequencing technologies, it was soon discovered that the genes encoding for these proteins were all related and diversified from a common ancestral gene. There are roughly a dozen families belonging to the TGF-β superfamily, and these can be divided into two major classes: the TGF-β-like class and the bone morphogenetic protein-like (BMP) class. The TGF-β-like class includes TGF-β *sensu stricto*, Lefty, Activin/Inhibin, and Myostatin/Gdf8. The BMP class includes Bmp2/4/Dpp, Bmp5–8, Bmp3, Gdf2, Gdf5–7, Vg1/Univin, ADMP, and Nodal. Besides being known for its roles in morphogenesis, TGF-β signaling, especially via Bmp2/4/Dpp, is also known for its role in dorsal-ventral patterning in both protostomes and deuterostomes (reviewed in [6]–[7]).

**Figure 1:**
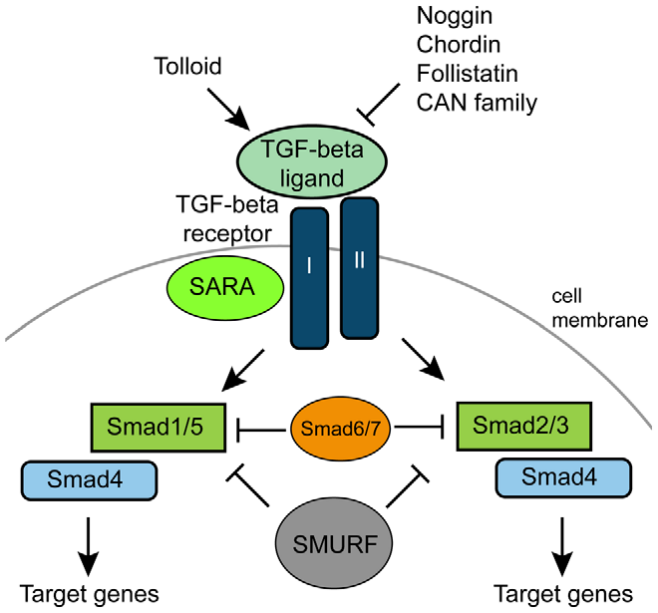
Basic overview of TGF-β signaling pathway. Binding of a ligand to a Type II receptor initiates signaling. The sequestering of a Type I receptor results in the activation of a Receptor-Smad (Smad1/5, Smad2/3). Together with the Co-Smad (Smad4), this complex enters the nucleus and activates the transcription of target genes. The pathway can be inhibited by extracelluar antagonists, or intracellularly via Inhibitor-Smad (Smad6/7) or the ubiquitin ligase SMURF.

The TGF-β precursor protein has three distinct regions: (1) the signal peptide, which targets it to the endoplasmic reticulum and secretion; (2) the propeptide, or the latency associated peptide; and (3) the mature peptide, which is cleaved from the precursor protein and is actively involved in signaling [8]. Whereas the mature peptide is highly conserved across different families, the propeptide is not. The mature peptide is cleaved by Furin, a convertase, at a dibasic arginine-X-X-arginine (RXXR) site [9]. The active peptide forms a hetero- or homodimer, which binds to a specific TGF-β Type II receptor (Figure 1) [3]. The Type II receptor then recruits a TGF-β Type I receptor and phosphorylates it via its serine/threonine kinase domain. Phosphorylated Type I receptors then phosphorylate (and thereby activate) receptor-associated Smad proteins (R-Smads), including Smad1/5 and Smad2/3 (For reviews, [10]–[11]). R-Smad proteins are composed of two main functional domains, the Mad-homology domains 1 and 2 (MH1 and MH2). Smad1/5 is associated with BMP-like signaling, while Smad2/3 is associated with TGF-β-like signaling. Inactive R-Smads are associated with the membrane via the Smad anchor for receptor activation (SARA) protein, which contains a zinc finger FYVE domain [12]. Activated R-Smads are released into the cytosol where they interact with the common-mediator Smad (Co-Smad, aka Smad4), and then become translocated to the nucleus. This heteromeric complex then regulates TGF-β target genes by interacting with transcription factors, including Fos/Jun and Myc, or co-activators, such as the Creb-binding protein (CBP) [13]. The MH1 domain is capable of interacting with DNA, while the MH2 domain interacts with Type I receptors and is involved with protein-protein interactions, such as R-Smad/Co-Smad binding.

Inhibition of TGF-β signaling can occur at multiple levels: extracellularly, cytoplasmically, and in the nucleus. Extracellularly, diffusible antagonists such as Chordin, Noggin, Follistatin and the CAN family (Cerberus/DAN/Gremlin) act as ligand traps, interfering with ligand binding to receptors [14]. In turn, the zinc metalloprotease Tolloid is capable of cleaving Chordin, thereby releasing BMPs to become active, showing that there are many levels of regulation involved with TGF-β signaling [15]. Besides cleaving Chordin, Tolloid also functions to cleave pro-collagens of the extracellular matrix [16], as well as other proteoglycans, some of which also are known to bind TGF-β ligands [17].

Intracellularly, the pathway can be inhibited at many levels. At the level of the receptors, FKBP12 can block Type I receptor phosphorylation by binding to the GS domain [18]. BAMBI, a pseudoreceptor, can prevent the Type I and Type II receptors from forming a receptor complex [19]. Pathway modulation can also occur via inhibitor-Smads (I-Smad, Smad6/7), which have an MH2 domain (like other Smads) and can bind to Type I receptors, interfering with R-Smad binding and phosphorylation [20]. I-Smads can also compete with R-Smad in binding with Co-Smads. Another intracellular regulator of TGF-β signaling is the Smad ubiquitin regulatory factor (SMURF), an E3 ubiquitin ligase that targets R-Smads for degradation [21]. SMURF can also be recruited by I-Smads to degrade Type I receptors at the membrane. TGF-β signaling is also regulated within the nucleus by the binding of co-repressors Ski/Sno [22]. These proteins recruit other repressors to block the activation of TGF-β target genes.

All levels of the TGF-β signaling pathway are highly conserved in metazoans, with pathway members present in all animals studied to date [23], [24]. Outside the metazoa, no TGF-β receptor or ligand has been discovered, so this pathway most likely evolved early in animal evolution. In the choanoflagellate, *Monosiga brevicollis*, an MH2 domain is present; however, it is unlike all known Smad proteins in that it is accompanied by a zinc finger domain [25]. Amongst the non-bilaterians (cnidarians, poriferans, the placozoan, and ctenophores), most of our knowledge regarding this pathway is gleaned from cnidarians [26]–[33]. Interestingly, this pathway has been implicated in axial patterning in cnidarians, similar to its role in dorsal-ventral patterning in bilaterians. Work in the sponge, *Amphimedon queenslandica*, has also shown that TGF-β signaling may be involved in axial patterning [34]. To date, there is nothing known about this pathway in the final group of non-bilaterians, the ctenophores. To better understand the evolution of this pathway, we need to be able to compare all the non-bilaterian taxa.

The ctenophore body plan and body axes are specified early in development. Developmental potential is segregated to different lineages; however, the exact molecules involved are unknown. Analysis of the genomic sequence of the lobate ctenophore, *Mnemiopsis leidyi*, allowed us to identify a near-complete TGF-β signaling pathway composed of nine ligands, four receptors, and five Smads, revealing that the core components are present in all metazoans studied to date. Notably absent are extracellular diffusible antagonists, including Chordin, Follistatin, Noggin, and CAN family members. We looked at the expression of these genes during ctenophore development and found expression of ligands to be differentially expressed along all three body axes (oral-aboral, tentacular, and sagittal). While we do not believe this pathway is necessarily specifying these axes, since they are expressed after the axes are already specified, we do believe they are involved with transducing earlier signals.

## Results

### Ligand diversity

**Figure 2:**
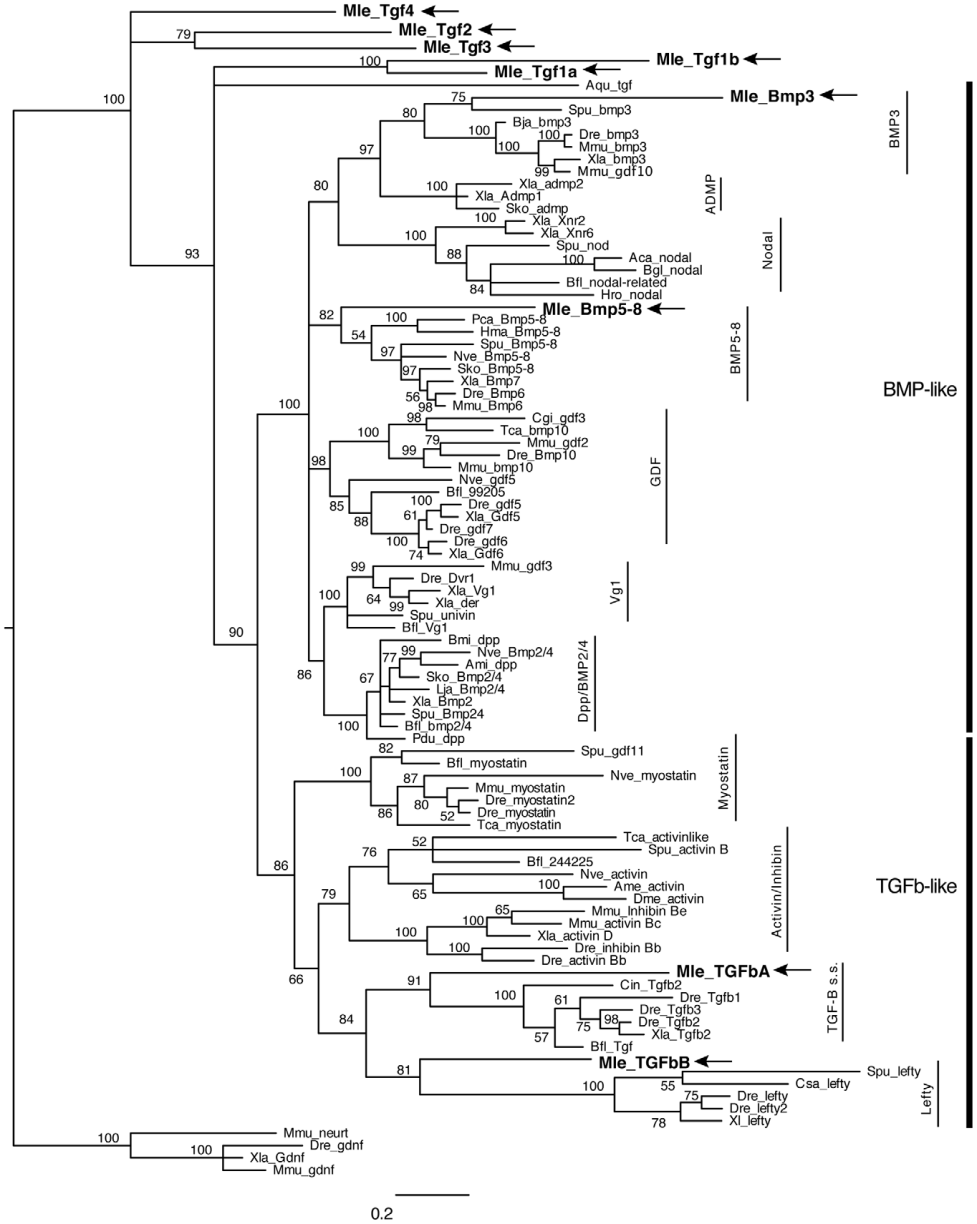
Bayesian analysis of TGF-β ligands. Analyses were performed using only the TGF-β peptide domain, with *Mnemiopsis* members bolded and marked by arrows. Representative taxa from deuterostomes, protostomes, and non-bilaterians were used (for full list of taxa, see Table S1). Four independent runs of five million generations were run using the “mixed” model, with the strict consensus tree shown. Nodes are labeled with posterior probabilities.

**Figure 3:**
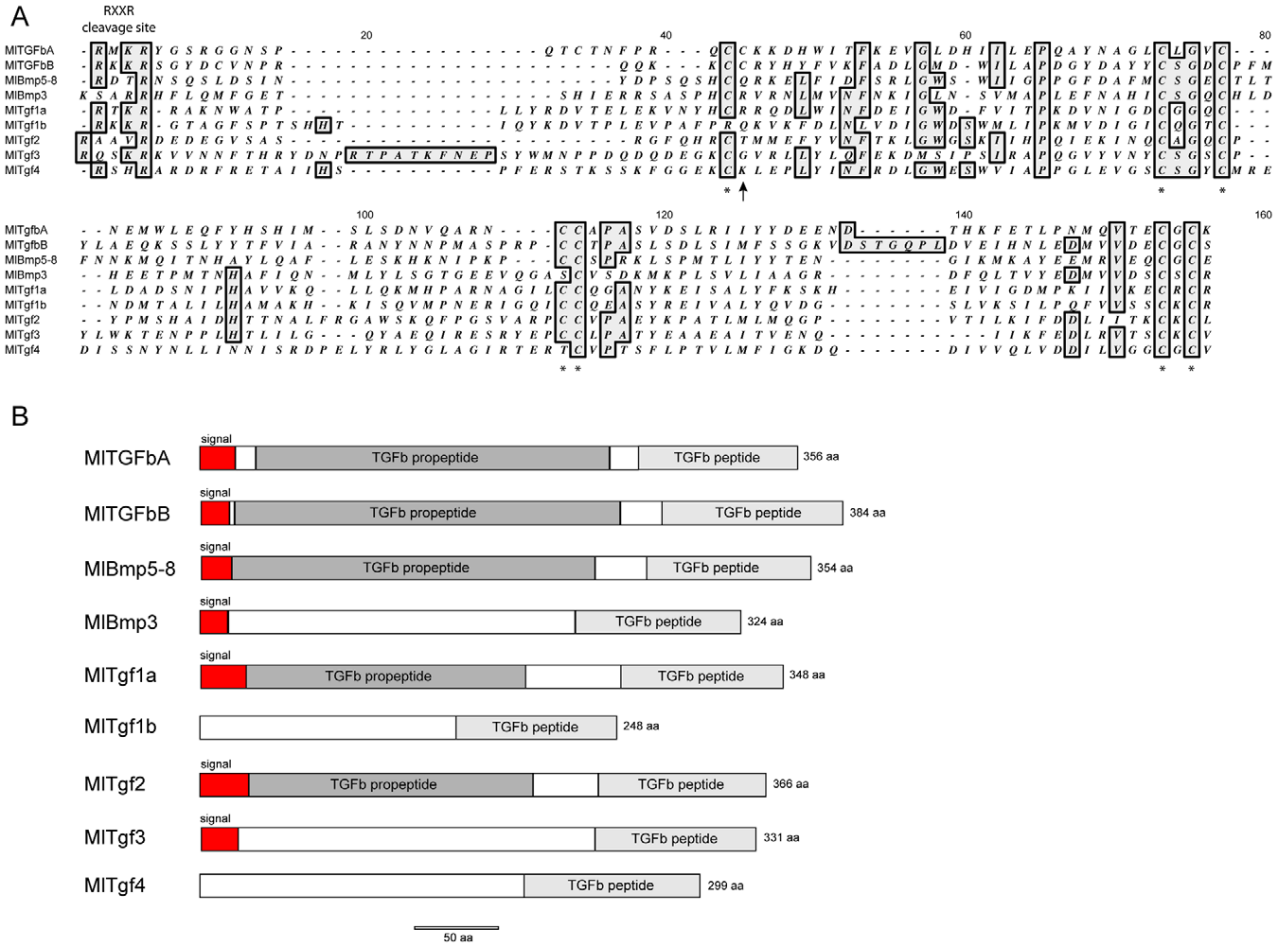
TGF-β protein structures and motifs. (**A**) Predicted amino acid sequences of the TGF-β peptide domain and flanking region. Adjacent to the peptide domain is the cleavage site, showing the conserved RXXR motif. Asterisks below the sequence mark the seven conserved cysteine residues. The arrow indicates the conserved cysteine found in TGF-β-like class of ligands. (**B**) Conserved protein domains of *Mnemiopsis* TGF-β ligands. The red boxes indicate signal sequences, while the other shaded boxes represent TGF-β propeptide and TGF-β peptide domains, as predicted by SMART.

Similar to the situation previously seen for the Wnt/β-catenin pathway, searches of the *Mnemiopsis* genome have revealed a near complete TGF-β signaling pathway (Table 1). We were able to identify and isolate nine putative TGF-β ligands, four receptors, and five Smads. The nine ligands include members of both the TGF-β-like and the BMP-like clades. Due to the relatively high divergence of the ctenophore sequences, only four could be placed in supported families by phylogenetic analyses: *MlTGFbA* and *MlTGFbB*, which are most closely related to TGF-β-like families TGF-β *sensu stricto* and Lefty (hence capitalized “TGF”), as well as *MlBmp3* and *MlBmp5–8* (Figure 2). However the posterior probability support is rather low (less than 95%), suggesting that there is a lack of phylogenetic signal in just the peptide domain sequence. When further analyses were run on the TGF-β-like clade using both the propeptide domain and the peptide domain, *MlTGFbA* and *MlTGFbB* end up as sister to the Activin+Myostatin grouping (data not shown); therefore, we do not think these genes are actually TGF*-β sensu stricto* or Lefty orthologs per se, but rather divergent members of the TGF-β-like clade. The other five ligands (*MlTgf1a*, *MlTgf1b*, *MlTgf2*, *MlTgf3*, and *MlTgf4*) group as sister to the other families (hence lower case “Tgf”). *MlTGFbA* and *MlTGFbB* both have eight cysteine residues, which are conserved in gene families of the TGF-β related clade (Figure 3A). *MlTgf1a*, *MlTgf3*, and *MlBmp5–8* have seven conserved cysteines, while *MlTgf1b*, *MlTg4*, and *MlBmp3* have only six. *MlTgf1b* is missing the first cysteine, while *MlTgf4* and *MlBmp3* are missing the fourth cysteine at position 113 in the alignment. Two of the genes appear to be relatively recent tandem duplications (*MlTgf1a and MlTgf1b*) since they group closely together and are located adjacent to each other on the same scaffold. It is likely that *MlTgf1b* is the result of a retroposition due to the fact that it is so closely linked to *MlTgf1a* and it does not contain any introns. The seven remaining genes are on separate contigs.

**Table 1:** TGF-β pathway members in *Mnemiopsis* genome.

Gene name	Mle Gene ID (Genbank Accession)	E value	Human hit
MlBmp5–8	ML218835 (JN380180)	3e-30	NP_001191.1: BMP2
MlTGFbA	ML102235 (JN380181)	9e-13	ABI48419.1: Myostatin
MlTgf3	ML048212 (JN380182)	3e-11	NP_003230.1: TGF-beta 3
MlTgf2	ML34871 (JN380183)	7e-12	AAH33585.1: Nodal
MlTGFbB	ML19322 (JN380185)	1e-24	ABI48386.1: Myostatin
MlTgf4	ML35889 (JN380185)	9e-13	NP_001001557.1: GDF6
MlBmp3	ML368915 (JN380186)	2e-12	AAH28237.1: GDF10
MlTgf1a	ML200252 (JN380187)	2e-10	NP_005802.1: GDF11
MlTgf1b	ML200253 (JN380188)	9e-7	NP_057288.1: GDF2
Smad6	ML19701 (JN380189)	1e-37	NP_005576.3: Smad6
Smad4	ML02191 (JN380190)	4e-72	NP_005350.1: Smad4
Smad1a	ML093050 (JN380191)	2e-128	NP_001120689.1: Smad9
Smad1b	ML01205 (JN380192)	1e-138	NP_005896.1: Smad9
Smad2	ML017743 (JN380193)	3e-149	NP_005893.1: Smad3
TgfRII	ML08593 (JN380194)	1e-51	AAH67417.1: Activin A receptor, type IIA
TgfRIa	ML082117 (JN380195)	2e-77	NP_004603.1: TGF beta receptor I
TgfRIb	ML131110 (JN380196)	2e-105	NP_004603.1: TGF beta receptor I
TgfRIc	ML046516 (JN380197)	7e-88	NP_004603.1: TGF-beta receptor I
SMURF	ML20687 (JN380198)	6e-158	NP_073576.1: SMURF2
Tolloid/BMP-1	ML016314 (JN380199)	3e-54	NP_036596.3: tolloid-like
Nomo	ML05901	5e-170	AAH65535: NOMO 1
HtrA	ML279621	5e-48	AAH11352.1: HTRA1
Furin	ML07022	0.00	EAW62576.1: proprotein convertase subtilisin
Jun	ML1541120	5e-22	CAG46525.1: JUN
Myc	ML004911	6e-10	1202343A: N-myc
MAX	ML1381	3e-08	NP_002373: max isoform a
CBP	ML274431	2e-154	AAC51770.1: CREB-binding protein
JNK	ML078937	7e-81	NP_620634.1: JNK1 beta1
	ML08261	2e-126	NP_002743.3: JNK2 alpha2
Ski/Sno	not detected		
Noggin	not detected		
Follistatin	not detected		
Chordin	not detected		
CAN family	not detected		
Fos	not detected		
SARA	not detected		

Homology searches using SMART [35] predicted signal peptides, TGF-β propeptides, and TGF-β peptides for *MlTGFbA*, *MlTgf2*, *MlTGFbB*, *MlBmp5–8*, and *MlTgf1a* (Figure 3B). For *MlBmp3* and *MlTgf3*, a signal peptide and TGF-β peptide are predicted, but the TGF-β propeptide is not. In the case of *MlTgf4* and *MlTgf1b*, only the TGF-β peptide is predicted. In the latter cases, the propeptides are missing or they are highly divergent and not detected by homology searches. The mature peptide cleavage site of RXXR is clearly present for all ligands, with the exceptions of possible modification for *MlBmp3* (KSAR), *MlTgf2* (RAAVR), and *MlTgf3* (RQSKR).

### Pathway members

**Figure 4:**
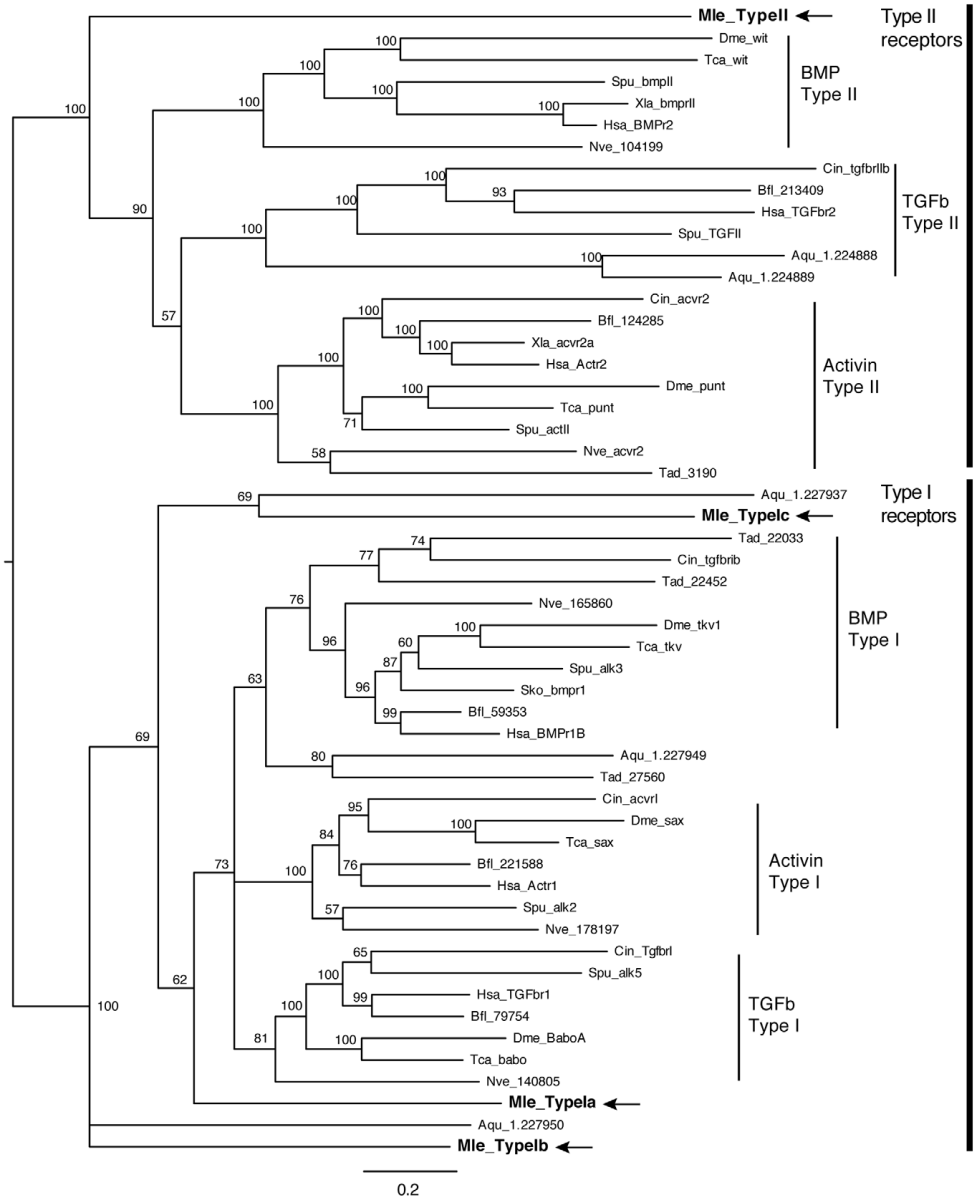
Bayesian analysis of TGF-β receptors. *Mnemiopsis* members are bolded and marked by arrows. Representative taxa from deuterostomes, protostomes, and non-bilaterians were used (for full list of taxa, see Table S1). Four independent runs of 5 million generations were run using the “mixed” model, with the strict consensus tree shown. Nodes are labeled with posterior probabilities.

There is a single Type II receptor (*MlTgfRII*) and three Type I receptors (*MlTgfRIa*, *MlTgfRIb*, and *MlTgfRIc*). All contain the extracellular receptor domain, the single pass transmembrane domain, and the intracellular serine-threonine kinase. Additionally, all three Type I receptors possess the glycine-serine repeat (GS region) adjacent to the kinase domain, an arrangement that is characteristic of Type I receptors. Phylogenetic analyses that included sequences of TGF-β receptors from representative metazoans show that, while there is strong support for the different subclasses (Type II: BmpRII/wit, TGF-βRII, ActivinRII/punt; Type I: BmpRI/tkv, ActivinRI/sax, TGF-βRI/babo), the *Mnemiopsis* receptors are not well supported in individual subclasses (Figure 4). Instead, *MlTgfRII* falls sister to all other Type II receptors (Figure 4). *MlTgfRIa* groups with the three Type I subclasses, while *MlTgfRIb* and *MlTgfRIc* are outside of these, grouping weakly with sponge genes.

**Figure 5:**
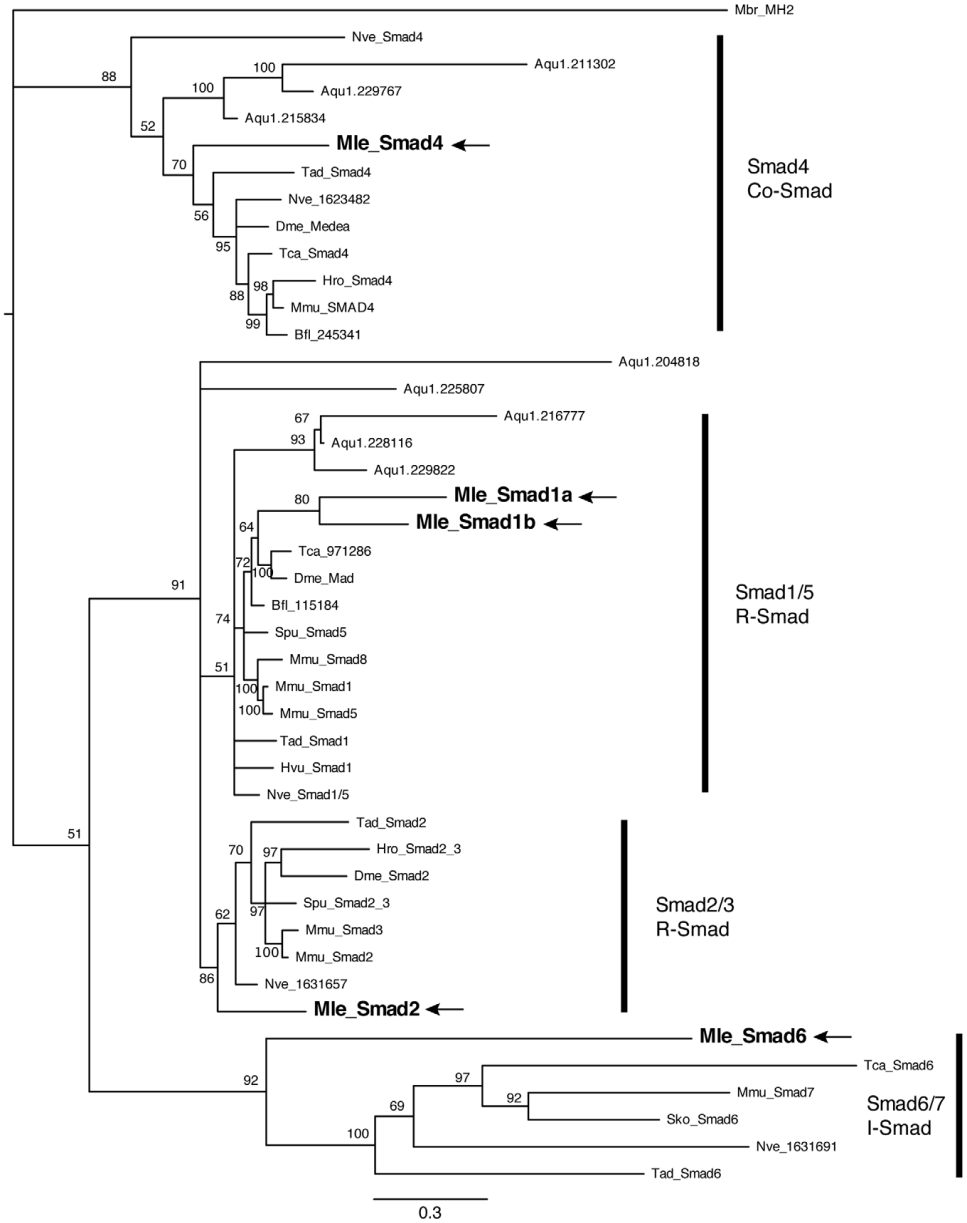
Bayesian analysis of Smad proteins. *Mnemiopsis* members are bolded and marked by arrows. Representative taxa from deuterostomes, protostomes, and non-bilaterians were used (for full list of taxa, see Table S1). Four independent runs of 5 million generations were run using the “mixed” model, with the strict consensus tree shown. Nodes are labeled with posterior probabilities.

We were also able to detect and isolate five Smad family members. Unlike the TGF-β receptors, these genes all grouped in moderately well-supported Smad families (Figure 5). There are three receptor Smads, two belonging to the Smad1/5 family (*MlSmad1a*, *MlSmad1b*) and one Smad2/3 (*MlSmad2*). There is a single Co-Smad (*MlSmad4*) and a single inhibitory or I-Smad (*MlSmad6*). *MlSmad4*, *MlSmad1a*, *MlSmad1b*, and *MlSmad2* have the predicted MH1 and MH2 domains, characteristic of Smad proteins. *MlSmad6* has the MH2 domain, as well as an amino terminal domain that resembles an MH1 domain. We were also able to identify and clone the E3 ubiquitin ligase SMURF, which can bind to receptor Smad proteins and target them for degradation, thereby inhibiting the cascade. Other intracellular components, including Jun, Myc, Max, CBP, and JNK, are present in the *Mnemiopsis* genome (Table 1). However we were not able to identify an ortholog of SARA, a protein that is involved with recruiting receptor Smads to the receptor [36]. There is also no apparent TGIF (transforming growth-interacting factor) protein; this homeodomain transcription factor acts with nuclear Smads as a co-repressor [37]. We were also not able to identify Ski/Sno or Fos.

Although *in silico* searches have discovered von Willebrand-type domains and Follistatin-like domains, we have not been able to find true Chordin, Noggin, Follistatin, or Gremlin orthologs, which are known diffusible antagonists of TGF-β signaling. Interestingly, we were able to identify a Tolloid gene (*MlTolloid*), which is known for enhancing signaling by cleaving Chordin, as well as other proteins. We also identified a Nodal Modulator (Nomo) ortholog, even though there is no true Nodal gene.

### Early TGF-β expression

**Figure 6:**
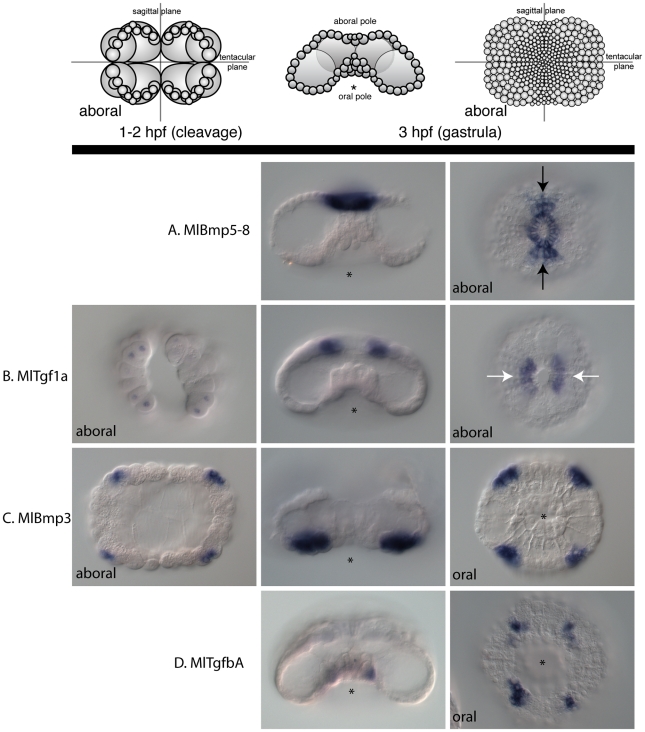
Early TGF-β mRNA expression. Four of the TGF-β genes are detected early in development, prior to and during gastrulation. The schematic at the top depicts the stages of embryos during cleavage and gastrulation, at 1–2 and 3 hours post fertilization (hpf), respectively. Embryos are lateral views, otherwise oral/aboral as stated. The asterisk marks the position of the blastopore. (**A**) *MlBmp5–8* expression in the aboral ectoderm, with more expression detected in the sagittal plane (black arrows). (**B**) *MlTgf1a* expression is detected in late cleavage stages around the nuclei of aboral micromeres. By gastrulation, the aboral expression remains, however there expression is primarily along the tentacular plane (white arrows). (**C**) *MlBmp3* is detected in four groups of ectodermal cells from early to mid-gastrulation. (**D**) *MlTGFbA* is detected in four groups of ectodermal cells just adjacent to the blastopore at gastrulation.

We examined the expression patterns of TGF-β and Smad genes during development. A set of TGF-β genes (*MlBmp5–8*, *MlBmp3*, *MlTgf1a*, and *MlTgfbA*) are expressed relatively early in development, just prior to and during gastrulation (Figure 6). These genes are expressed in staggered domains along all three body axes. *MlBmp5–8* is expressed in the most aboral region, surrounding the aboral pole in cells that will form the apical organ (Figure 6A). There is also more extensive staining in cells along the sagittal plane than the tentacular plane (see black arrows). *MlTgf1a* begins expression prior to gastrulation at about two hours post fertilization (hpf) in 12–16 micromeres at the aboral pole (Figure 6B). Unlike all other genes studied here, expression begins confined to the nuclei or the perinuclear region. At gastrulation, these cells give rise to portions of the aboral pole, primarily in the tentacular plane (see white arrows). At this time, expression is cytoplasmic, so it is not clear what the significance is of the earlier nuclear expression or how it changes to the cytoplasm. In this stage of development, expression overlaps with that of *MlBmp5–8*. *MlBmp3* is expressed in four groups of ectodermal micromeres towards the oral pole at the onset of gastrulation (Figure 6C). This expression is very transient since transcripts cannot be detected in later stages of development. Finally *MlTgfbA* is expressed in ectodermal micromeres around the blastoporal opening (Figure 6D). These genes are expressed in staggered ectodermal domains along oral-aboral axis, as well as differentially in the tentacular and sagittal planes.

### Late TGF-β expression

**Figure 7:**
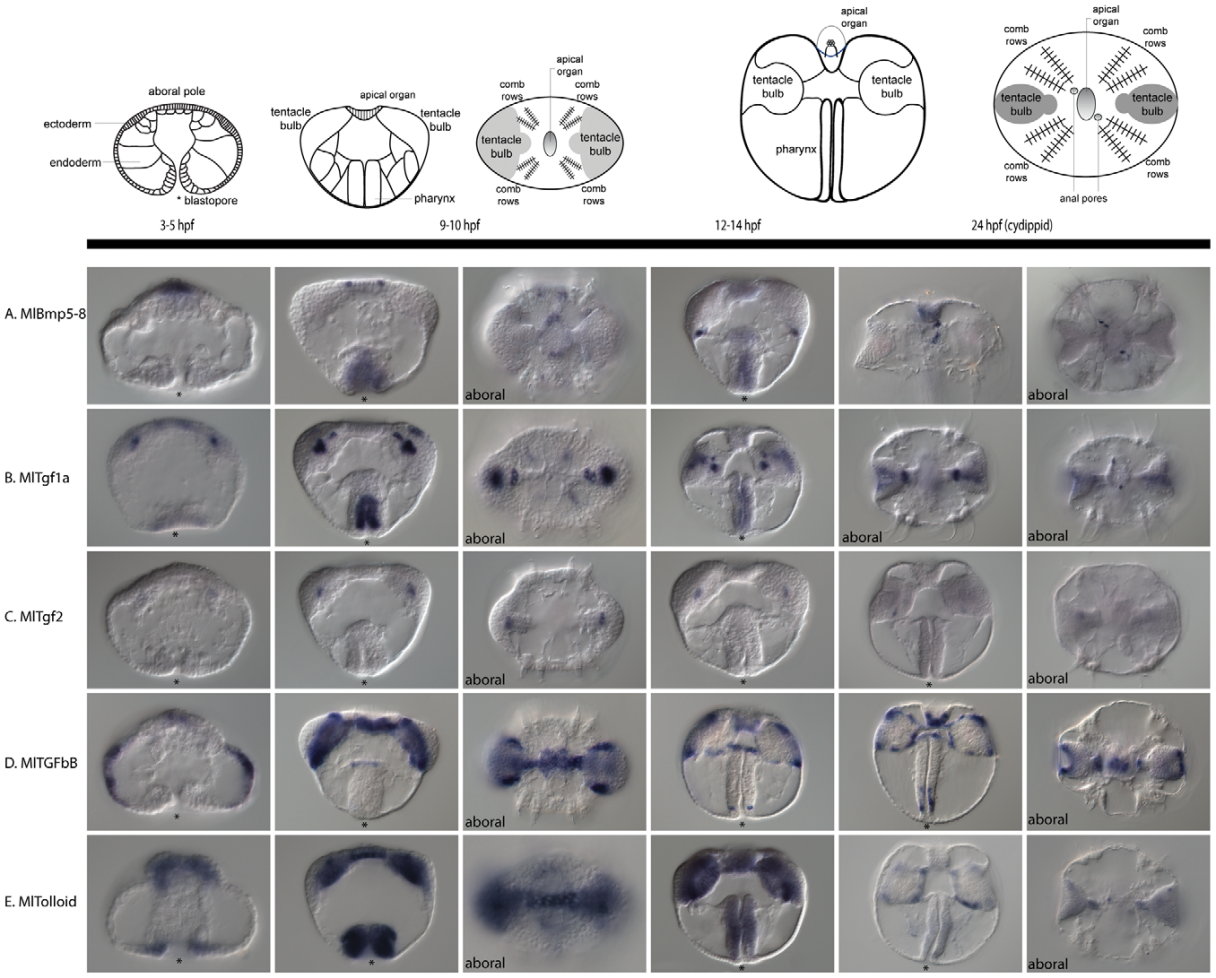
Late TGF-β mRNA expression. *MlBmp5–8*, *MlTgf1a*, *MlTgf2*, *MlTGFbB* and *MlTolloid* are detected during later stages of development. The diagram at the top depicts the stages of development in the columns below, identifying some of the major features and structures. Views are lateral, unless otherwise specified as oral or aboral. The asterisks marks the position of the blastopore or mouth. (**A**) *MlBmp5–8* expression in the aboral ectoderm and later in the invaginating pharynx. The aboral expression later becomes part of the apical organ and the anal canals. There is also an additional domain of expression in the tentacle bulbs. (**B**) *MlTgf1a* is expressed in parts of the tentacle bulbs, pharynx, and apical organ. (**C**) *MlTgf2* is expressed faintly in part of the tentacle bulbs, similar to that of *MlTgf1a*, however by cydippid stages, expression is barely detectable. (**D**) *MlTGFbB* is expressed after gastrulation in a fairly complex pattern. There are expression domains at the oral and aboral ends of the pharynx. There is also expression in parts of the tenacle bulbs and in the apical organ. (**E**) *MlTolloid* is expressed around the blastopore and later in the pharynx, as well as in mesodermal derivatives, in transtentacular muscle and parts of the tentacle bulbs.

In later developmental stages, we were able to examine the expression of four TGF-β genes (*MlBmp5–8*, *MlTgf1a*, *MlTgf2*, and *MlTgfbB*). The primary areas of expression for these genes are within the tentacle bulb and in the pharynx (Figure 7). *MlBmp5–8* is expressed in a few cells of the apical organ, which correspond to the cells of the early expression domain and faintly in the pharynx (Figure 7A). It is also expressed in the tentacle bulb, in the most oral region. In cydippid stages, this pharyngeal and tentacular expression is not present, and there is only expression in the apical organ and anal pores. *MlTgf1a* is primarily expressed in two regions of each tentacle bulb, a larger region in the central part of the bulb and a smaller region (2–4 cells) closer towards the apical organ (Figure 7B). There is also expression in parts of the pharynx. In cydippids, there is an additional expression domain in two small regions of the apical organ in the most sagittal areas. *MlTgf2* is expressed faintly in the tentacle bulb that overlaps with *MlTgf1a* expression (Figure 7C). *MlTgfbB* has the broadest expression domain, which includes a large portion of the tentacle bulb, the oral and aboral extremes of the pharynx, the muscle cells connecting the tentacle bulbs, and the floor of the apical organ. Although we were able to clone the remaining TGF-β ligands (*MlTgf1b*, *MlTgf3*, and *MlTgf4*) from mixed stage cDNA, we were not able to detect their expression via *in situ* hybridization. We also analyzed the expression of the metalloprotease, *MlTolloid*, and found that it is expressed after gastrulation around the blastopore and in cells that have entered the blastocoel (Figure 7E). In later stages, it is expressed along the entirety of the pharynx, as well as in a the tentacle bulbs and transtentacular musculature, which overlaps with the expression of *MlTGFbB*. However expression is not detected in the apical organ, and in cydippid stages, expression levels appear to be downregulated, in comparison to earlier in development.

### TGF-β receptor and Smad expression

**Figure 8:**
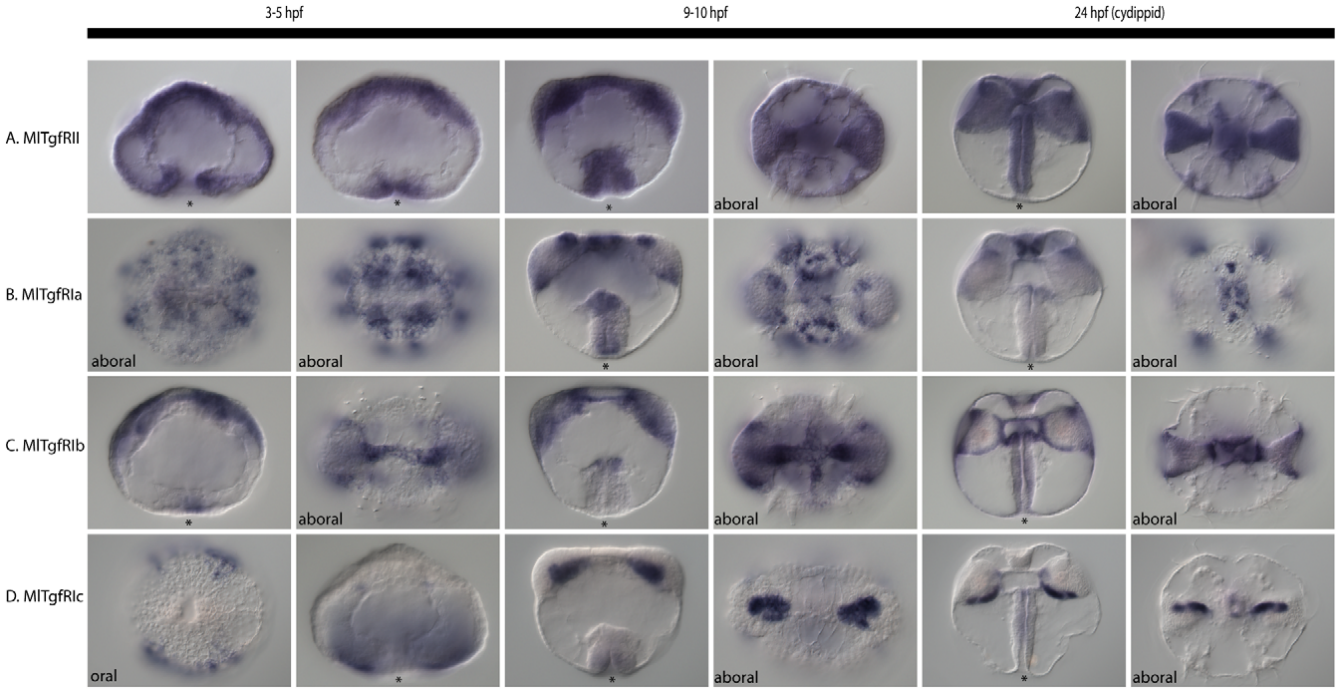
TGF-β receptor expression patterns. Expression of TGF-β receptors through development, from gastrulation (3 hpf) to cydippid (24 hpf). Views are lateral unless otherwise specified, and asterisks mark the position of the blastopore or mouth. (**A**) *MlTgfRII*, the lone Type II receptor, is expressed ubiquitously from egg through cypdippid stages. (**B**) *MlTgfRIa* is expressed in the aboral ectoderm as well as in the pharynx. The aboral ectoderm expression is confined to the developing comb rows and apical organ. (**C**) *MlTgfRIb* is detected in the pharynx, as well as in mesodermal derivatives. Cydippid expression is confined to parts of the tentacle bulb, as well as the endodermal part of the gut. (**D**) *MlTgfRIc* is expressed in the ectoderm, more towards the oral pole. Late expression is confined to parts of the tentacle bulbs.

The lone Type II receptor is expressed ubiquitously from egg to cydippid stage (Figure 8A). Contrastingly, the three Type I receptors are expressed in relatively non-overlapping regions. *MlTgfRIa* is expressed initially at gastrulation in aboral ectodermal tissue, then in later stages in the apical organ, in cells around the comb rows, and faintly along the entire pharynx (Figure 8B). *MlTgfRIb* is expressed in the muscle that connects the tentacle bulbs, in the most aboral part of the pharynx, in the outer regions of the tentacle bulb, and possibly also in the endoderm (Figure 8C). *MlTgfRIc* is expressed initially in ectoderm towards the oral pole (Figure 8D). This expression fades and a later expression domain shows up in the mesoderm, which forms part of the tentacle bulb.

**Figure 9:**
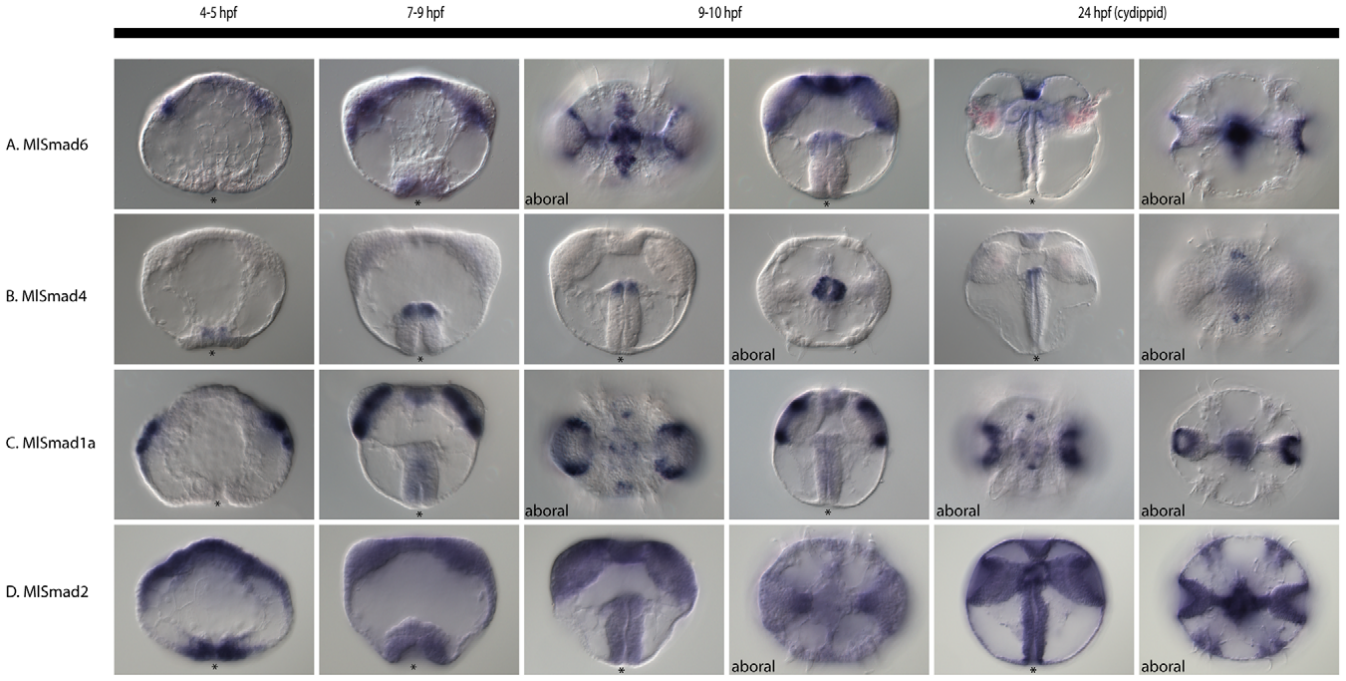
Smad expression patterns. mRNA expression of *Mnemiopsis* Smad genes during development. All views are lateral, unless otherwise specified. The asterisk marks the position of the blastopore or mouth. (**A**) *MlSmad6*, the I-Smad, is expressed in mesodermal derivatives of the tentacle bulb, as well as in the apical organ. (**B**) *MlSmad4*, the Co-Smad, is expressed in a discrete domain of the pharynx at the ectoderm-endoderm boundary. There is also late expression in the apical organ in four spots. (**C**) *MlSmad1a*, an R-Smad, is expressed in parts of the tentacle bulb and apical organ. (**D**) *MlSmad2*, another R-Smad, is expressed ubiquitously from egg to cydippid.

*MlSmad6*, which is the I-Smad, is expressed in the mesoderm, apical organ, and the aboral part of the pharynx (Figure 9A). In cydippids, only the apical organ expression remains, as well as the outer portion of the tentacle bulb. *MlSmad4*, the Co-Smad, is only expressed in the aboral part of the pharynx, which forms the boundary of the ectodermal and endodermal portion of the gut (Figure 9B). In the cydippid stage, there is an additional staining in the apical organ in a few cells in the sagittal plane (Figure 9B). *MlSmad1a* is expressed in a somewhat similar region in the tentacle bulb and apical organ as the receptor *MlTgfRIa* (Figure 9C). We were not able to detect the expression of *MlSmad1b* via *in situ* hybridization. *MlSmad2* is expressed ubiquitously from egg to cydippid (Figure 9D).

### TGF-β inhibitor SB431542

**Figure 10:**
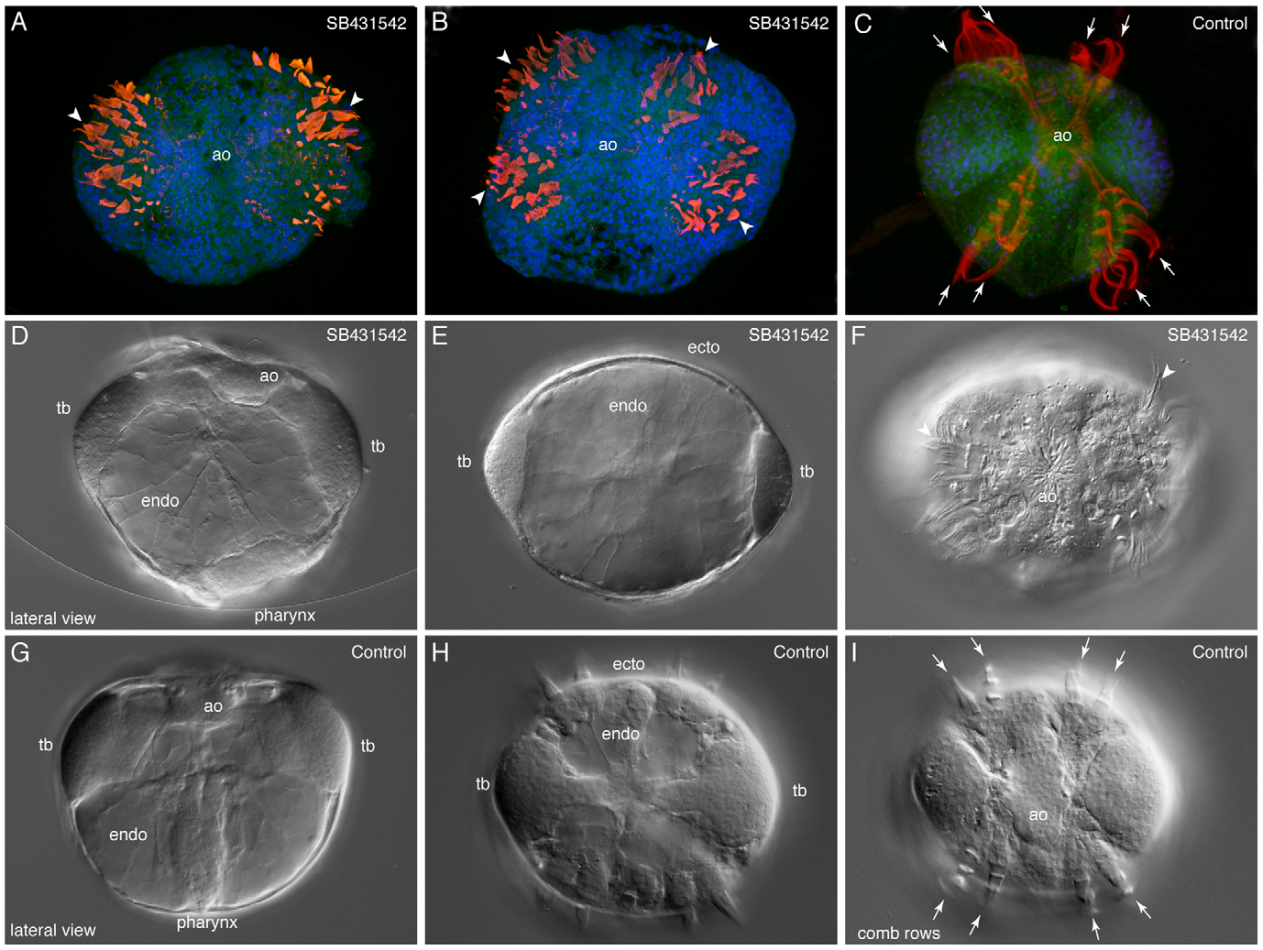
SB431542 treatment during *Mnemiopsis* development. Effects of TGF-β inhibitor, SB431542, at 12 hours post fertilization. (**A,B, D–F**) are treated embryos, while (**C, G–I**) are controls. (**A–C**) Confocal projections of embryos stained with anti-tyrosinated tubulin (red) showing the cilia, Alexa-488 phalloidin (green) showing cell borders, and Hoechst 33342 (blue) showing the nuclei. All are aboral views, with the apical organ (ao) in the center. The white arrowheads point to individual comb plates, while the arrows in (C) show the eight comb rowsC. (**D–I**) are live embryos imaged under DIC. (**D**) Lateral view of SB431542-treated embryo, showing that pharynx has not invaginated (compare to (**G**)), and the tentacle bulbs (tb) have formed but are smaller in size. The apical organ appears normal. (**E**) Aboral view of the same embryo, mid-focal plane, again showing the smaller tentacle bulbs. The ectoderm (ecto) and endoderm (endo) both appear normal. (**F**) Aboral and surface view, showing the disorganized comb plates (arrowhead), compared to the eight comb rows (arrows) in the control (**I**).

To better understand the function of TGF-β signaling, we used the drug SB431542 (CAS 301836-41-9) to interfere with the signaling pathway. It has been shown in other animals to inhibit the activity of alk5/TGF-β Type I receptors [38]. Treatment of *Mnemiopsis* eggs at concentrations less than 25 µM resulted in normal cydippids. Treatment between 25–50 µM resulted in consistent morphological defects. Rather than forming eight rows of comb plates, the combs appear to be clustered in two to four groups (Figure 10A–C). In addition to being in clusters, the combs are also not organized in rows, such that they do not beat synchronously. In addition, there is a thickening of the pharyngeal ectoderm, but it does not invaginate inward (Figure 10D). There are also thickenings where the tentacle bulbs are; however, they appear to be smaller than usual, and tentacles never grow out from these bulbs. The apical organ forms normally, and the ectoderm and endoderm also appear to be relatively normal.

In addition to the morphological phenotypes, development is delayed slightly when compared to wild type animals. Raising the animals in SB431542 for longer than 12 hours results in death. When embryos are treated after gastrulation (3–4 hpf), the embryos develop normally, implying that there is a window during which signaling is active. While there is no true alk5/TGF-βRI receptor, the gene that is phylogenetically most closely related to this receptor is *MlTgfRIa*. It is likely that the effects that we see are authentic, as this gene is expressed in the forming comb rows from gastrulation onward, as well as in the invaginating pharynx.

## Discussion

### Evolution of the TGF-β signaling pathway

Both the Wnt/β-catenin pathway and the TGF-β pathway likely evolved early in metazoan evolution, with the core components present in all animals studied to date [23]–[25]. However unlike the Wnt pathway, where some of the proteins (or, at a minimum, specific domains) are found in non-metazoans, including beta-catenin-like and frizzled-like proteins, nearly all of the TGF-β pathway genes are metazoan-specific. There are no known ligands or receptors found outside the metazoa, although serine/threonine kinase domains similar to those in TGF-β receptors are found in other eukaryotes. Additionally, there are no Smad genes in any other eukaryote, although there is a single Smad-like MH2 domain in the choanoflagellate, *Monosiga*. This domain is coupled with a C2H2 zinc finger, which is unlike all other Smad genes [25]. Searches of the recently sequenced genomes of the eukaryotes *Salpingoeca rosetta* and *Capsaspora owczarzaki* have also not revealed any TGF-β ligands, receptors, or Smads. Therefore, the origin of this pathway may have been a key innovation in metazoan evolution.

Within the metazoa, the diversity and total number of TGF-β receptors and Smads are relatively constant (Table 2). One exception to this observation is the sponge (*Amphimedon*), which has multiple duplications of Smad genes. Another exception is the vertebrates (and teleosts, in particular), which have an expanded set of Smads and receptors, most likely due to lineage-specific genome duplications. In comparison, the number of TGF-β ligands is much more variable. This is consistent with the hypothesis that there are more constraints on intracellular relative to the extracellular components of the signaling pathway [5]. The Smads and intracellular regions of the TGF-β receptors can be utilized for multiple purposes and in response to various ligands and signals. On the other hand, the ligands themselves are not so highly constrained, which might explain why there are so many more ligands than receptors and why the sequences of the ligands are much less conserved than those of the receptors and Smads. It is possible that ligands diversified and were co-opted for multiple developmental processes, while the intracellular components were reused.

**Table 2:** Non-bilaterian TGF-β pathway components.

	*Mnemiopsis*	*Amphimedon*	*Trichoplax*	*Nematostella*
TGF-β ligands - Total	9	8	5	6
BMP-like	2	0	4	4
TGF-β-like	2	2	1	2
Unclassified	5	6	0	0
TGF-β receptors - Total	4	5	4	5
Type I	3	3	3	3
Type II	1	2	1	2
Smads - Total	5	10	4	4
Smad4	1	3	1	1
Smad1/5	2	3	1	1
Smad2/3	1	2	1	1
Smad6/7	1	0	1	1
Unclassified	0	2	0	0

**Figure 11:**
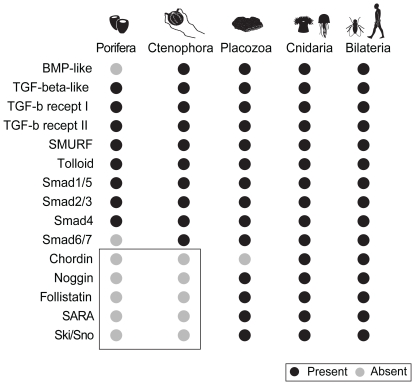
Summary of presence and absence of TGF-β components. The rows contain the different TGF-β components. The columns represent the four early-branching lineages of the Metazoa, plus the Bilateria. Each row represents the presence (black dot) or absence (grey dot) of a particular component in the corresponding lineage. The box shows the absences shared by Porifera and Ctenophora. Data is compiled from genomic data of *Amphimedon queenslandica* (Porifera), *Mnemiopsis leidyi* (Ctenophora), *Trichoplax adhaerens* (Placozoa), *Nematostella vectensis* and *Hydra magnipapillata* (Cnidaria), and *Drosophila melanogaster* and *Homo sapiens* (Bilateria).

While the core components of ligand-receptor-downstream mediators appear to have co-evolved, the addition of antagonistic ligand regulation appear to have arisen later. Similar to the sponge, *Amphimedon*, the *Mnemiopsis* genome does not contain any of the known diffusible antagonists (Figure 11). Since both *Amphimedon* and *Mnemiopsis* possess Tolloid orthologues, the ancestral function of this metalloprotease and BMP enhancer must have targeted proteins other than Chordin. Noggin, CAN family members and Follistatin are present in cnidarians, *Trichoplax*, and bilaterians, while Chordin is only found in only cnidarians and bilaterians. Chordin-like genes have been found in *Amphimedon* and *Trichoplax*; however, these lack the CHRD domain of true chordin genes [24]. In addition, both SARA and Ski/Sno are present in cnidarians, *Trichoplax*, and bilaterians, but absent from ctenophores and sponges. Assuming that there was not a secondary loss, these were also later additions to the signaling pathway, giving more support to the early branching position of ctenophores and sponges. Interestingly, there is no obvious relationship between morphological complexity and signaling pathway complexity, at least in the non-bilaterians. In comparison to sponges and *Trichoplax*, ctenophores are much more morphologically complex, yet they have a similar (if not simpler) TGF-β signaling pathway complement.

What is also surprising is that the ligand complements of the non-bilaterians are not conserved. Many of the ctenophore and sponge ligands do not group with major bilaterian subclasses (Figure 2) [24], [25], but the cnidarian, *Nematostella vectensis*, and placozoan, *Trichoplax adhaerens*, sequences do (Figure 2 for *Nematostella*; *Trichoplax* not shown). However, comparing just BMP-like and TGF-β-like classes, we see that all non-bilaterians have at least one TGF-β-like gene, while all non-bilaterians (except sponges) have a BMP-like gene, suggesting that this radiation also occurred early in animal evolution. Whether the unclassified ctenophore and sponge genes represent extremely divergent remnants of other classes or if they are just novel genes that arose in these lineages remains to be seen. Interestingly, if the ligand orthology does hold to be true, these would be the first orthologs of Bmp3, TGF-β sensu strictu, and Lefty identified outside the deuterostomes. However due to the low support values, we have doubts as to whether this is the case.

### Expression patterns

The expression patterns of the TGF-β ligands *MlBmp3*, *MlBmp5–8*, *MlTgf1a*, and *MlTgfbA*, are highly suggestive of a role in axial patterning. They are all expressed relatively early in development (at gastrulation) and are expressed differentially along all three body axes. They are expressed in ectodermal micromeres, with *MlBmp5*–*8* expressed the most aborally, followed by *MlTgf1a*, *MlBmp3*, and *MlTgfbA* most orally. Additionally, there is differential expression along the tentacular and sagittal planes, with *MlTgf1a* expressed mainly along the tentacular plane, while *MlBmp5–8* is expressed more along the sagittal plane, although there is some overlap with *MlTgf1a*. However, experimental embryological evidence indicates that even at this early stage of development, the axes are already specified [39], [40], so these ligands may be transducing earlier signals. Possibly low levels of ligand expression during early cleavage stages are undetectable by *in situ* hybridization, or perhaps there are maternal proteins that are localized in the egg and early embryo. As early as the four-cell and eight-cell stages, factors that specify different cell types, including comb plates and photocytes, are localized to different lineages [39]. Whether these determinants are proteins or mRNAs is not known, however we have yet to see differential mRNA expression at these early stages, suggesting that these factors could be maternal proteins. Additionally, at these stages, only the Type II receptor (*MlTgfRII*) and a TGF-β-like Smad (*MlSmad2*) are expressed. None of the Type I receptors are expressed at this stage, and neither is the Co-Smad, *MlSmad4*. The Type I receptors are not detected until 4–5 hours post fertilization, about an hour after the earliest ligand expression. It is also possible that these ligands are initiating signals via Smad-independent pathways, such as the MAP kinase, Rho-like GTPase, or PI3K/AKT pathways [13], [41].

One interesting aspect of the expression patterns is that, while the lone Type II receptor is expressed uniformly, the three Type I receptors are expressed in non-overlapping domains. Assuming protein distribution is similar, this would suggest the specificity of the response to ligands is dependent on which Type I receptor is expressed. *MlTgfRIa* is expressed predominantly in the ectoderm, specifically in the forming comb rows and in the apical organ. Meanwhile, *MlTgfRIb* is expressed broadly in the mesoderm and endoderm, while *MlTgfRIc* is expressed in putative mesoderm of the tentacle bulb.

The observation that *MlSmad4*, the only Co-Smad, is detected in only a small region of cells, the pharynx at the ectoderm-endoderm boundary and a few cells of the apical organ, implies that it might not be necessary for signaling in other areas. Perhaps the R-Smads can function independently or with other factors to activate transcription of target genes. There is evidence from mammalian systems showing that Smad2/3 can bind to non-Smad proteins, including IKKα [42] and TIF1γ [43], to illicit signaling independently of Smad4. Whether this is the case in Mnemiopsis, and what exactly the binding partners are remains to be seen. Functional work is needed to determine whether the *Mnemiopsis* R-Smads are even capable of binding the Co-Smad. The I-Smad, *MlSmad6*, is expressed in many areas that are overlapping with other Smads and Type I receptors (*i.e.*, in the mesoderm, apical organ, ectoderm-endoderm boundary, and tentacle bulb). This suggests that there is both active signaling and highly complex regulation in these regions, as both activators and inhibitors of the pathway are co-expressed in the same cells and regions. Given that these are discrete areas of the developing embryo/larva, the observed expression patterns suggest that TGF-β signaling may be important for germ layer specification or differentiation. For example, the apical organ is highly innervated and the primary sensory structure, and the tentacle bulb, where there is co-expression of activators and an inhibitor, is the site of putative stem cells for tentacle growth. The tentacle bulbs are regions of continual growth, suggesting that TGF-β signaling is also involved in proliferation and cell cycle regulation. In addition, the ligands *MlBmp5–8*, *MlTgf1a*, *MlTgf2*, and *MlTGFbB* are all expressed in regions of the tentacle bulb, suggesting this is an important signaling center of the developing embryo. It is likely that *MlTolloid* is also important for *MlTGFbB* function because of their highly overlapping expression domains. It is possible that *MlTolloid* is playing a role in cleavage and activation of this ligand, similar to its role in vertebrates and flies [44]–[46].

The results of our experiments with the TGF-β inhibitor SB431542 suggest that there is also a role of TGF-β signaling in comb row organization and morphogenesis. *MlTgfRIa* is expressed in the developing comb rows and is the most similar receptor to alk5/TGF-βRI, the known target of SB431542 [38]. The onset of MlTgfRIa expression (at gastrulation, 2.5–3 hpf) is within the window of sensitivity to SB431542. When exposed to the inhibitor, the comb plates still form at the correct time and display similar morphology, but they are not separated into eight rows and not organized in the same orientation. However, as is the case with any pharmaceutical inhibitor, there is a chance of non-specific effects, so further experiments such as injection of a morpholino antisense oligonucleotide designed against *MlTgfRIa* is necessary to ensure the drug is acting as we hypothesize. Recently, morpholino to the T-box gene, *brachyury*, has been shown to specifically inhibit its function during development by blocking pharyngeal invagination [47]. Since we obtained a similar pharyngeal defect using SB431542, it is possible that *brachyury* is a target of TGF-β signaling, similar to both frog and the chick *brachyury* that are direct targets of Activin-like signaling [48], [49]. Thus TGF-β signaling could be playing a role in ctenophore pharyngeal morphogenesis by activating *brachyury*. Exactly how it is mediating comb row organization has yet to be determined.

In conclusion, the TGF-β signaling pathway was present and most likely active early in metazoan evolution. With few components present in extant non-metazoans, it is highly probable that the emergence of this pathway was a key innovation in the transition to multicellularity in the metazoan ancestor. While a Smad-like gene is present in the choanoflagellates, there is very little similarity of TGF-β signaling ligands and receptors outside of the metazoa. From expression studies here, it appears that TGF-β signaling is active in the ctenophore embryo. However, it is unlikely that this pathway is involved in early axis specification. The earliest expression of any TGF-β ligand is just prior to gastrulation, after the embryonic axes are already specified. The staggered expression patterns of the ligands at gastrulation is suggestive that TGF-β signaling is responding to earlier signals. It remains to be seen what these early signals are, but it is possible that proteins for components of this pathway (and other key pathways) could be maternally loaded.

## Materials and Methods

### Genome search and phylogenetic analyses

We utilized the *Mnemiopsis* draft genome, which was previously sequenced using 454 and Ilumina sequencing and assembled onto scaffolds [50]. This sequence data was compiled into 10,106 scaffolds (scaffold N-50 of 123 kb), which corresponds to a physical coverage of approximately 50×. Searches for TGF-β pathway components are similar to those in searches for Wnt pathway components [51], using a reciprocal Blast approach. Cnidarian and bilaterian gene orthologs were used in tblastn searches of the *Mnemiopsis* genome assembly. Putative positive matches were then aligned to orthologs from other organisms. Alignments were performed using MUSCLE (www.drive5.com/muscle) and then corrected by eye. For TGF-β ligands, only the mature peptide domain was used in phylogenetic analyses. For TGF-β receptors, we used the extracellular receptor, transmembrane, and intracellular kinase domains. For the Smad proteins, the MH1 and MH2 domains were used. All alignments are located in the Supporting Information files (Text S1, S2, S3). For all trees, we used Mr.Bayes3.2 [52], using the ‘mixed’ model with four independent runs of five million generations, with trees sampled every 100 generations. Consensus trees and posterior probabilities were calculated once the stationary phase was obtained.

### Gene isolation and expression studies

Genes of interest were isolated using RACE PCR (Clontech), with all verified sequences being deposited into GenBank (JN380180–JN380199). *In situ* hybridizations were as previously described [53]. Full-length or partial-length sequences, ranging in size from 800 bp to 2 kb, were used to transcribe digoxigenin-labeled RNA probes. We detected these probes using an alkaline phosphatase-conjugated digoxigenin antibody, utilizing the substrates NBT and BCIP to then detect the alkaline phosphatase activity (Roche). Specimens were mounted in 70% glycerol, viewed under a Zeiss AxioSkop, and imaged using an AxioCam.

### SB431542 treatments

Embryos were obtained from adult animals in Woods Hole, MA during the summers of 2009 and 2010 as previously described [53]. Following collection, they were treated with the pharmacological agent SB431542, a potent inhibitor of TGF-β signaling that blocks Type I receptor activity [38]. We started soaking one to four-cell stage embryos at concentrations from 25–50 µM in 24-well plates (30–50 embryos per well, approximate volume 1.0 ml). Treated embryos were immersed in SB431542 through their entire development and kept in the dark as much as possible. They were monitored periodically and fixed at 9–12 hours post fertilization (hpf).

### Antibody staining and confocal microscopy

Embryos were fixed for antibody staining in 4% paraformaldehyde and 0.02% glutaraldehyde, as described previously [53]. Following fixation, embryos were removed from their membranes by gentle pipetting. They were then washed with PBS plus 0.2% Triton (PBT) and then placed in blocking buffer (5% goat serum) for one hour. They were then incubated with anti-tyrosine tubulin (Sigma, T9028) overnight at 4°C. Following six 30-minute washes with PBT, they were then incubated with a secondary antibody, goat anti-mouse conjugated to Alexa-594 (Invitrogen, Molecular Probes). After an overnight incubation, they were again washed with PBT six times for 30 minutes. In the last wash, they were also incubated with Alexa-488 phalloidin (Invitrogen, Molecular Probes) and Hoechst 33342 (Invitrogen, Molecular Probes). Following two 5-minute washes in PBS, they were then mounted on a slide and imaged using a Zeiss 710 confocal microscope. Images were processed using Zen software (Zeiss) and Volocity (Improvision) to create 3D image reconstructions of confocal sections.

## Supporting Information

Table S1 **Taxa used in phylogenetic analyses.** The first column lists the different phyla, the second column lists the species, and the third column lists the abbreviation used in the phylogenetic trees and alignments.
(DOC)

Text S1 **Amino acid alignment of TGF-β ligands.** Shown here are only the mature peptide sequences for taxa shown in Table S1, which was used to generate the tree in Figure 2. They were aligned using Muscle, then corrected by hand.
(NEX)

Text S2 **Amino acid alignment of TGF-β receptors used in** **Figure 4** **.** Shown here are the extracellular receptor, transmembrane, and intracellular kinase domains.
(NEX)

Text S3 **Amino acid alignment of Smad proteins used in** **Figure 5** **.** Shown here are the MH1 and MH2 domains.
(NEX)
