# Supplementary material for: Evolution of the TGF-β Signaling Pathway and Its Potential Role in the Ctenophore, Mnemiopsis leidyi
Source: PLoS One. 2011 Sep 8;6(9):e24152. doi: 10.1371/journal.pone.0024152 (PMC3169577; doi:10.1371/journal.pone.0024152)
Supplement: Table S1 — Taxa used in phylogenetic analyses. The first column lists the different phyla, the second column lists the species, and the third column lists the abbreviation used in the phylogenetic trees and alignments. (DOC) [file pone.0024152.s001.doc]

Table S1. Taxa used in phylogenetic analyses

| **Phylum** | **Species** | **Abbr** |
| --- | --- | --- |
| Choanoflagellata | *Monosiga brevicollis* | Mbr |
| Ctenophora | *Mnemiopsis leidyi* | Mle |
| Porifera | *Amphimedon queenslandica* | Aqu |
| Placozoa | *Trichoplax adhaerens* | Tad |
| Cnidaria | *Acropora millepora* | Ami |
|  | *Hydra magnipapillata* | Hma |
|  | *Nematostella vectensis* | Nve |
|  | *Podocoryne carnea* | Pca |
| Annelida | *Capitella teleta* | Cte |
|  | *Platynereis dumerilii* | Pdu |
| Mollusca | *Aplysia californica* | Aca |
|  | *Biomphalaria glabrata* | Bgl |
|  | *Crassotrea gigas* | Cgi |
| Arthropoda | *Bombyx mori* | Bmo |
|  | *Drosophila melanogaster* | Dme |
|  | *Tribolium castaneum* | Tca |
| Echinodermata | *Strongylocentrotus purpuratus* | Spu |
| Hemichordata | *Saccoglossus kowaleskii* | Sko |
| Chordata | *Branchiostoma floridae* | Bfl |
|  | *Branchiostoma japonicum* | Bja |
|  | *Ciona intestinalis* | Cin |
|  | *Ciona savignyi* | Csa |
|  | *Danio rerio* | Dre |
|  | *Halocynthia roretzi* | Hro |
|  | *Homo sapiens* | Hsa |
|  | *Lethenteron japonicum* | Lja |
|  | *Mus musculus* | Mmu |
|  | *Xenopus laevis* | Xla |
